# Primary Liver Cancers: Connecting the Dots of Cellular Studies and Epidemiology with Metabolomics

**DOI:** 10.3390/ijms24032409

**Published:** 2023-01-26

**Authors:** Shoma Berkemeyer

**Affiliations:** Faculty of Agricultural Sciences and Landscape Architecture, Osnabrueck University of Applied Sciences, Am Kruempel 31, 49090 Osnabrueck, Germany; s.berkemeyer@hs-osnabrueck.de

**Keywords:** hepatocellular carcinoma, cholangiocarcinoma, hepatoblastoma, metabolomics, WNT/β-catenin pathway, P13K/AKT/mTOR pathway, lipid, TCA

## Abstract

Liver cancers are rising worldwide. Between molecular and epidemiological studies, a research gap has emerged which might be amenable to the technique of metabolomics. This review investigates the current understanding of liver cancer’s trends, etiology and its correlates with existing literature for hepatocellular carcinoma (HCC), cholangiocarcinoma (CCA) and hepatoblastoma (HB). Among additional factors, the literature reports dysfunction in the tricarboxylic acid metabolism, primarily for HB and HCC, and point mutations and signaling for CCA. All cases require further investigation of upstream and downstream events. All liver cancers reported dysfunction in the WNT/β-catenin and P13K/AKT/mTOR pathways as well as changes in FGFR. Metabolites of IHD1, IDH2, miRNA, purine, Q10, lipids, phosphatidylcholine, phosphatidylethanolamine, acylcarnitine, 2-HG and propionyl-CoA emerged as crucial and there was an attempt to elucidate the WNT/β-catenin and P13K/AKT/mTOR pathways metabolomically.

## 1. Introduction

Liver cancer is the third largest cause of cancer deaths worldwide [1]. Globally, it is the sixth most common cancer [2]. The global incidence is increasing [2]. The primary liver cancer in adults is hepatocellular carcinoma (HCC), which accounts for about 80–90% of cases [2]. The second most common liver cancer is Cholagniocarinoma (CCA), which accounts for 10–15% of the cases [2]. Remnant cases of liver cancer are hepatocellular cholangiocarcinoma, angiosarcoma and hepatoblastoma (HB) [2]. The primary liver cancers in children are HB and HCC [2,3]. HB accounts for about 70% of the cases and HCC accounts for about 27% of the cases [2,3]. Each type and sub-type of liver cancer has its own etiology.

Genetic investigation of liver cancers attributes damage and mutations to specific genes in cancer etiology. An example is mutation A235G in the catenin beta 1 (*CTNNB1*) gene (refer to Abbreviations), which led to upregulation of the gene in HB and HCC [3,4]. Another example is upregulation of C-X3-C motif chemokine (*CX3CL1*) gene, due to repeated missense mutation on exon 3 [3]. This leads to substitution of the amino acid alanine by glycine in protein translation, with changes in inflammation and immunity metabolites [3]. These connections between genes and systemic metabolites are tedious and require investigation. Between large-scale population and epidemiological studies [1,2] and cellular studies [3,4] metabolomics offer a means of investigation, which incorporates systemic adjustments of maintenance of homeostasis, systemic and cellular feedback loops and compensatory mechanisms. Metabolomics is increasingly offering a means to connect basic science with clinical epidemiology, and to personalize diagnosis, prognosis, therapy and follow-up [5]. It offers the advantage of large-scale testing of metabolites either, invasively, in blood and tissue samples, or, non-invasively, in urine and saliva samples [5], facilitating simultaneous observation of a range of metabolites. Thus, this review will update our current understanding on three major liver cancers, HCC, CCA and HB, using metabolites, in a preliminary effort to connect the dots of cellular studies with clinical epidemiology. 

## 2. Current Understanding of Liver Cancer Trends, Etiology and Correlates

The liver is an organ of active yet differentiated metabolism. For example, glucose uptake, formation of glutamine and xenobiotic metabolism are located in the perivenous area [6]. Bile acid synthesis, urea synthesis, oxidative phosphorylation and glucose output are located in the periportal area [6]. Amathieu et al. [5] proposed that blood and urine metabolome ought to be considered as outcome of liver metabolism and, thus, the phenotype. This is so since the liver reports the summation of acute, chronic and acute, moving on to chronic metabolism in any human body [5].

### 2.1. Current Epidemiological and Moleular Understanding of Hepatoblastoma (HB)

HB is a rare embryonal tumor, most commonly occurring in childhood [2]. The cause of the increasing incidence of HB is largely unknown [2,6,7]. The rising incidence is attributed to increasing survival rates of premature and low birth weight neonates [5,6], and genetic diseases of Wilms’ tumor, familial polyposis coli, paraneoplastic syndrome and Beckwith–Weidman syndrome [5,6,7]. A population-based study reported a dose-dependent increase in relative risk of HB, i.e., a relative risk of 1.56 at birthweight of <2500 g, a relative risk of 3.37 at birthweight between1500–2500 g and a relative risk of 17.18 at birthweight of <1500 g compared to reference normal birthweight >2500 g [8]. This US-based study [8] was corroborated with similar results from Nordic countries, Japan, China, the United Kingdom and a confirmatory US population register study [6].

Tumor presentation in young children is accompanied by failure to thrive, weight loss and enlarged abdominal mass along with tumor mass characteristics of tissue necrosis, hemorrhage and calcifications [7]. At mRNA transcriptomic analysis, Cairo and colleagues [9] determined HB-subtypes C1 and C2 groups. Overexpression of mature hepatocyte markers of genes of cytochrome P450 family, glutamate-gated chloride channels and rhamnogalacturonase (*CYP1A1*, *CYP2E1*, *GLUL* and *RHBG*) characterized C1 group [9]. Upregulation of hepatic stem cells and proliferation markers of alpha fetoprotein, aurorakinase B, brain expressed X-linked, baculoviral IAP repeat containing 5, mitotic checkpoint serine/threonine-protein, cyclin-dependent kinase 1, discs large associated protein 5, delta like non-canonical notch ligand 1, histone deacetylase 2, insulin-like growth factor 2, maternally expressed 3, necdin, nucelophosmin 1, paternally expressed genes and protein epithelial cell adhesion molecule (*AFP*, *AURKB*, *BEX1*, *BIRC5*, *BUB1*, *CDC2*, *DLG7*, *DLK1*, *HDAC2*, *IGF2*, *MEG3*, *NDN*, *NPM1*, *PEG3*, *PEG10* and *TACSTD1*) characterized C2 group [9]. Epigenetic DNA methylations and histone modifications, and genomic changes were used for HB identification as well. Epigenetic profiling classified two HB subtypes of epigenetic cluster A and epigenetic cluster B, suggesting first molecular risk stratification [10]. DNA sequencing studies reported mutations of the Wnt/β-catenin almost exclusively affecting the *CTNNB1* gene, which encodes β-catenin [3,4,10]. Additional gene mutations reported were *AXIN1*, *AXIN2* [9]. Long deletions of *CTNNB1* exons 3 and 4 were reported exclusively in fetal HB [3,10]. Genetically, HB is a “simple” tumor, since it has an average 2.9 mutations per tumor [7]. Several pathogenetic molecular pathways have been described for HB. The primary ones are WNT β-catenin (WNT/β-catenin) pathway, Hippo pathway, MYC pathway, Hedgehog pathway, Nuclear factor, erythroid 2 like bZIP transcription factor 2-Kelch like ECH associated protein 1 (NFE2L2/KEAP1) pathway, hepatocyte growth factor-mesenchymal-epithelial transition factor (HGF/c-MET) pathway, intracellular signaling phosphoinsositide-3-kinase/protein kinase B AKT of AGC-kinase family/mammalian target of rapamycin (P13K/AKT/mTOR) pathway, substance P and the neurokinin-1 receptor (SP/NK-1R) pathway and insulin-like growth factor (IGF) pathway [7]. Of all the pathways, the WNT/β-catenin and NFE2L2/KEAP1 pathways appear most crucial and suggest epigenetic regulation for the development, histologic appearance, and tumor malignancy [11]. Epigenetic regulation, thus, might offer possibilities in management or treatment of an embryonal tumor, warranting generational studies, especially epigenetic changes in mothers and embryonal tumors in progeny. 

A study reports cases of HB and HCC in a clinical study from infancy to adulthood [12]. Five cases showed methylmalonic aciduria indicating mitochondrial dysfunction, impairment of tricarboxylic acid cycle, oxidative stress and possible effects of oncometabolities [12]. Eichenmüller and colleagues corroborated this in another study [4]. Supplementation of hepatocytes with selenium mitigated oxidative stress-dependent repression of apolipoprotein A–I expression by suppression of Nuclear factor kappa-light-chain enhancer of activated B-cells protein complex (NF-kappaB) pathway [13]. The NFE2L2/KEAP1 pathway activation with *CTNNB1* mutations with loss of genomic stability and telomerase-reverse-transcriptase (TERT) promoter was reported for aggressive HB with HCC features [4]. Based on cancer registry studies, HB was reported in 213 from 14.805 cases of cancers (172/9.352 at an age of diagnosis of 0–10 years; 41/5.453 at age of diagnosis of 11–20 years) in Scandinavia [14]. 

### 2.2. Current Epidemiological and Moelcuar Understanding of Cholagniocarinoma (CCA)

Classically, CCA was reported in two major forms, the intrahepatic (iCCA) and extrahepatic (eCCA) forms. The consensus statement on CCA reports two additional current forms used, i.e., the perihilar CCA (pCCA) and distal CCA (dCCA) [15]. The Consensus statement discourages the use of the term eCCA, because of the inherent difficulty in distinguishing eCCA and iCCA, and because of combination subtypes, such as combined HCC-CCA (cHCC-CCA) tumor [15]. Thus, classification in terms of iCCA, pCCA and dCCA emerges prescient [15]. 

CCA risk factors include hepatolithiasis, liver fluke infection, chronic inflammation of the bile ducts called cholangitis and exposure to thorotrast [2,15,16,17]. De Martel and colleagues reported that parasitic sub-type of iCCA, which is endemic to Far Asia, led the International Agency for Research on Cancer to classify the liver fluke parasites, Clonorchis sinensis, Opisthorchis felineus and Opisthorchis viverrini, as class I carcinogens [2,17]. However, it is the incidence and mortality of the non-parasitic iCCA which is rising globally [15,16]. The global mortality rate of CCA, year 2012, was low (less than two deaths per 100,000 people) in Argentina, Chile, Brazil, Colombia, Puerto Rico, Venezuela and Mexico [15]. North America, Germany, Benelux, Scandinavia, South Europe, Israel, Australia and New Zealand reported mortality between two and four deaths per 100,000 people [15]. Far Asia, i.e., Thailand, China and South Korea, had an incidence of greater than six per 100,000 people and Taiwan had greater than four (excepting Japan, mortality statistics currently not reported for Far Asia) [15]. In Europe, Austria reported a high mortality rate of 4.04. Germany and UK reported moderate mortality rates of 3.2 and 3.1, respectively, which were slightly higher than other European countries [15]. All countries reported an increase in mortality and incidence over the past ten years [15]. Only Japan reported a reduction in mortality from a very high mortality rate of 6.52 in 2002 to a lower, but still very high mortality rate of 5.85 in 2012 [15]. Risk factors, such as hepatitis, smoking cigarettes, diabetes, alcohol consumption, nonalcoholic fatty liver disease, hypertension and liver cirrhosis are under investigation for non-parasitic CCA; half the cases of iCCA are idiopathic [2,15,16,17]. 

The main genetic mutations identified for CCA were in the genes of *BRAF*, *EGFR* and *KRAS* [18]. Additional mutations include the genes *ATM*, *BRCA1*, *BRCA2*, *CCND1*, *CDKN2A* and *TP53* [15]. Gene amplification of *MYC* and *METLL1* was also reported [15]. Epigenetic regulation seems pertinent as well and includes: (a) de-ubiquitination of BRCA1 associated protein 1 (BAP1) and Switch/Sucrose non-fermentable (*SWI-SNF)* complex (AT-rich interaction domains (*ARID1A*, *ARID1B*, *ARID2*), *PBRM1*, *SMARCA2*, *SMARCA4*, *SMARCAD1*), (b) histone (de-)methylation (*KDM4A*, *KDM5D*, *KDM6A*, *KDM6B*, *KMT2C*, *MLL2*, *MML3*) and (c) nicotinamide adenine dinucleotide phosphat NADP metabolism (Isocitrate dehydrogenase (IDH1 and IDH2)) [15,19]. The epigenetic changes, predominantly via *IDH1* and *IDH2* mutations, have been a genetic breakthrough leading to clinical observation of accumulation of oncometabolite 2-hydroxyglutrate (2-HG) and a gene fusion of *FGFR2* with various partners [19,20], such as, genes *BICCI1*, *MGEA5*, *PPHLN1* and *TACC3* [15,19]. Kinase signaling includes the reports of genes *BRAF*, *ERBB1-3*, *FGFR1-3*, *KRAS*, *PIK3CA*, *PTEN*, *SMAD4* and *STK11*, immune dysregulation include those of the Janus kinase signal transducer and activator of transcription (JAK-STAT3) signaling pathway and dysregulation of Notch signaling, fusions of *FGFR2* and *PRKCA-PRKCB*, and WNT-CTNNB1 and Hippo pathways [15,19,20,21]. 

CCA pathogenesis is complex, multistep process with increasing genomic and epigenetic changes combined with dysregulation of multiple signaling pathways (refer Table 1), where point mutations, copy number variations and chromosome fusions altering gene expression have been indicated [21]. CCA arises from cholangiocytes (biliary epithelial cells), while hepatic progenitor cells (HPC) and mature hepatocytes have been also suggested [22]. Sia and colleagues [23] found that undifferentiated somatic stem cells can differentiate to hepatocyte precursors, cholangiocyte precursors or a mixed subgroup of cHCC-CCA precursors; even so, the liver in comparison to other organs of the body has a slow turnover rate of several months and a hepatocyte life span of about 200 to 300 days [23]. So far, the mixed cHCC-CCA has shown greater aggressiveness and worse survival outcomes [24]. The mixed form cHCC-CCA is classified into five molecular subtypes depending on gene mutation: KRAS, ARID1A, TERT promoter, TP53 and IDH1/2 [24]. The common molecular subtypes in CCA and HCC reported for Asians were similar to Caucasians; the subtype C1 was reported to be driven by PLK1 and ECT2 and the C2 subtype linked to obesity, T-cell infiltration and bile acid metabolism [25]. 

### 2.3. Current Epidemiological and Molecular Understanding of Hepatocellular Carcinoma (HCC)

The most common liver cancer is the HCC type and the most common risk factors encompass the diseases of civilization relating to life-style factors, such as obesity, excess consumption of alcohol, cigarette smoking diabetes mellitus and nonalcoholic fatty liver disease [2,26,27]. Other risk factors are hemochromatosis and infections of hepatitis virus, predominantly Hepatitis B and Hepatitis C [2,26,27]. The transition from liver malfunction, clinically observable as steatosis progressing to fibrosis, cirrhosis, and eventually, in certain cases, to HCC is under physiological and molecular investigation. HCC is difficult to diagnose early, and late-stage diagnosis limits disease prognosis [28]. HCC seldom shows hyper-vascularity, indicating that it can survive in nutrient-poor environments, with growing indication that the tumor microenvironment probably maintains cancer stemness via tumor-initiating cells [29]. 

Low-glucose stress activates the PERK-mediated unfolded protein response, leading to expression of activating transcription factor 4 (ATF4), which binds to fucosyltransferase 1(FUT1) promoter, driving HCC [29]. Conversely, blocking FUT1 in cancer cells restricted HCC tumor initiation, indicating therapy options [29]. MicroRNA23a (miRNAs) was the most prominent miRNA in HCC [28], the overexpression of which indicated non-response in patients with HCC to drug sorafenib [28]. Not only glucose, but also fat metabolism has been indicated in HCC, and has been proposed to use the WNT/β-catenin pathway [30]. In the canonical WNT/β-catenin pathway, in the absence of Wnt-signal, β-catenin undergoes degradation. With Wnt-signal, i.e., WNT binding to frizzled or class F of G protein-coupled receptors (FZDs) and low density lipoprotein receptor-related protein (LRP5/6), there is an accumulation of β-catenin in cytoplasm and nucleus [30]. In the noncanonical pathway, the Wnt-pathway is under two negative feedback controls via ring finger protein 43 (RNF43) and cell-surface transmembrane E3 ubiquitin ligase zinc and ring finger (ZNRF3), and mutations in both promote HCC [30], in support of WNT-signal. RNF43 and ZNRF3 are both E3-ubiquitin ligases which ubiquitinate the cytoplasmic loops of the FZDs receptors, which induce rapid endocytosis (endo-lysosomal degradation) [31]. R-spondin 2 (RSPO), ligands of LGR4-5-6 receptors, also interact with RNF43 and ZNRF3, reversing the promotion by FZDs of WNT-signal strength and duration [32]. Mutations in RNF43 and ZNRF3, thus, have been reported in HCC [30,31,32] but also in liver fibrosis [33]. Likewise, mutations in RNF43 and ZNRF3 have been reported in altered lipid metabolism, specifically in unsaturated fatty acids, acyl-CoA biosynthesis and non-alcoholic steatohepatitis (NASH) [30]. The missing link between liver lipids, dietary fat supplementation and related liver regeneration is under investigation and the current literature [30,31,32,33] suggests that an ongoing impaired liver regeneration could probably accumulate, with cumulative malfunction over time. The pathophysiological observation of disease progression from steatosis, fibrosis, cirrhosis, and probably, eventually, to HCC [28] could become increasingly understandable, probably from the diseases of civilization etiology [2,26,27]. 

Tumor-infiltrating lymphocytes (TILs) and peripheral blood lymphocytes (PBLs) are two major HCC associated immune responses, and patients in advanced stage HCC showed poor immune response [34]. Tumor-induced immune suppression encompassed lower response to recalling antigens, reduced proliferative T-cell responses, loss of cytokine production, defective signal transduction in T-cells and natural killer (NK) cells [34], and increased apoptotic CD8^+^ T-cells in PBLs [35]. An increased population of regulatory T-cells (Tregs) was associated with invasiveness of HCC; Foxp3-expressing Tregs have been found to be co-opted by tumor cells and escape immune surveillance [36,37]. Jiang and colleagues [37] demonstrated that TILs, which were previously regarded only to eliminate tumor cells, could through Tregs trigger chronic inflammation, supporting tumorigenesis and metastasis [37]. The population of Tregs in HCC (other cancers too) increased on the expression of long noncoding RNA (lncRNA), specifically the lnc-EGFR, which bound to EGFR and thereby stabilized it and sustained EGFR activation [37]. This led to changes in RAS/ERK/AP1 signaling, leading to Treg differentiation, cytotoxic T-lymphocyte inhibition and HCC development via immunosuppression in HCC [37]. Mitogen-activated protein kinase (MAPK) consisting of the RAS/RAF/MEK/MAPK pathway (also known as RAS/ERK/AP1) has been also reported for HCC [38,39]. Alterations in the cytoplasmic signaling pathways produce a flow of mitogenic signaling without the stimulation by receptors of MAPK and this route plays a central role in about 25% of human cancers [38,39].

## 3. An Integrative Metabolomics Approach to Liver Cancers of Hepatoblastoma (HB), Cholangiocarinoma (CCA) and Hepatocellular Carcinoma (HCC)

Metabolomics is an omics-method of quantitative measurement of multiple parameters in living systems downstream to genomics, transcriptomics and proteomics, thus allowing a comprehensive assessment of net metabolic changes [5]. In augmentation of direct hepatic metabolomics, molecular phenomics linking hepatic transcriptomics and plasma and urine metabolomics have been also used in combination to understand, elucidate, predict, search causation, and where possible, effect diagnosis, treatment and prognosis [40]. Table A1 in Appendix A indicates that the Serin/Threonin-Kinase B-Raf, known as BRAF, of the RAS-MAPK pathway, and FGFR group of genes might be common observations to hepatic steatosis and liver cancer as per the current literature.

### 3.1. An Integrative Metabolomics Approach to Hepatoblastoma (HB)

Table 2 reports a range of metabolites that were reported in individual cases with HB [12]. Salient observations were a reduced activity of the Vitamin B_12_-dependent mitochondrial enzyme of methylmalonyl-CoA-mutase (MMUT), corresponding to high levels of plasma and urinary levels of metabolite methylmalonic acid [12]. Reports of the expression of β-catenin [12] indicated the involvement of the Wnt/β-catenin pathway [7]. Reduced expression of glutamine synthetase with lower reticulin probably indicates the Wnt/β-catenin pathway additionally [7]. Reduced tricarboxylic acid cycle (TCA), and thus, mitochondrial dysfunction, was indicated by reduced levels of succinyl-CoA [41], due to dysfunction of MMUT, thus accumulation of propionyl-CoA, which further inhibited pyruvate dehydrogenase complex (PDC) [41], additionally lowering mitochondrial efficiency. Excessive production of 2-methylcitrate from accumulating propionyl-CoA additionally inhibited enzymes of the TCA [42]. Jungermann and Kietzmann [43] elucidated that the normal physiological gene expression in hepatocytes was modulated by oxygen. In cultures under arterial oxygen tension, glucagon-induced activity of phophoenolpyruvate carboxykinase (PCK1) was higher compared to venous oxygen tension, followed by the glucagon-dependent increase in PCK1 transcription, with the abundance of its mRNA, at maximum arterial tension [43]. Glucokinase activity, induced by insulin, was at its maximum under perivenous oxygen tension [43]. Perivenous hypoxia leads to damaged hepatocytes due to increased ethanol in the perivenous zone; the free-radical-scavenging glutathione and glutathione peroxidase were located in the periportal area, thus exposing the perivenous area of liver disproportionately to prooxidants. Alcohol-induced liver disease occurs primarily in the perivenous zone [6], currently attributed to perivenous expression of ethanol-degrading enzymes ADH and CYP2E1, and the newer hypothesis of higher cytotoxicity of the perivenous occurring Kupffer cells [43]. Zonal differences in liver function have been reported [6]. Subsequent to increased oxidative stress in liver oncogenesis, prooncogenic signaling pathways were triggered, which in turn led to autophagy [44], nuclear factor ᴋ-B signaling [45] and hypoxia inducible factor 1-alpha, mitogen-activated protein kinase/ERK [46] and phophoinositide-3-kinase/AKT or P13K/AKT/mTOR pathways [11,46]. Blocks in TCA lead to the accumulation of intermediate metabolites of TCA, which function as oncometabolites in assessing dysfunction in liver metabolism [12]. Propionyl-CoA, one such metabolite, is a known modifier of histone acetylation [47]. Three other oncometabolites with their acidurias were reported, being fumarate, succinate and 2-HG, the latter of which appears increasingly in diagnostic and therapeutic contexts [12].

### 3.2. An Integrative Metabolomics Approach to Cholagniocarinoma (CCA)

Metabolic profiling of CCA reported increased 7β bile acid and decreased biliary phosphatidylcholine (PC) [48] (refer Table 2). Inhibition of hepatic PC impaired the secretion of very low-density lipoprotein, which is related to the development of fatty liver and impaired liver regeneration [49]. Banalis and colleagues [15] reported the use of serum, urine, bile and saliva as promising biomarkers in the coming years and report measurement of cell-free DNA or resistome (cfDNA) in liquid biopsies, which mirror tumor aggressiveness and size. Bile miRNA, especially miRNA21, proteins and cytokines, such as cytokeratin-19 (CYFRA 21-1), MMP, osteopontin, periostin, IL-6, fascin, EGFR, mucin 1 (MUC1), MUC4 and p27 were investigated in metabolite profiling [15]. The current molecular understanding of CCA (refer Section 2.2) identifies mutations in IDH1 and IDH2 as groundbreaking in CCA [19,20,21]. Mutations in *KRAS* [18,24] and *TP53* [15,24] were reported with shorter outcome survival and increased tumor recurrence rate, associated with higher levels of protein biomarkers EGFR, MUC1, MUC4, and fascin expression and lower levels of p27 expression, in addition to higher expression of miRNA21 [15]. Since 50% of CCAs consist of mutations or amplifications or fusions, such as, *IDH1*, *IDH2*, *BRAF*; *FGFR*, *HER2*, *PIK3CA*, *MET*, etc., metabolomic measurements of these could improve personalized care [15]. 

A range of inflammatory cytokines and chemokines, CXCL12/SDF-1, HMGB1, IL-6, TGF-β and TGF-α, have been reported in association with CCA [21]. In addition, hormones, such as, adrenomedullin, prostaglandin E2 and 17β-estradiol, and growth factors, such as, of the EFG-like family, FGF-19 and HGF, have been reported as metabolites in CCA [21]. Among the cytokines, TGF-β1 and TGF-β2 trigger SAMD dependent and independent cascades, specially *SMAD4* triggering of Notch, WNT-CTNNB1 and Hippo pathway [15,19,20,21]. TGF-β is markedly upregulated on liver injury, and is a tumor promoter at later stages of liver cancer, offering an understanding of probable common etiology of CCA and HCC or cHCC-CCA [21], over and above the stemness of hepatic cell development [22], with reports of greater tumor aggressiveness of the cHCC-CCA types [23]. 

TNF-α functions physiologically as a proinflammatory cytokine, responsible for innate immunity and surveillance against cancer. In CCA etiology, assessment of TNF-α is varied; it promoted cancer cell invasiveness by MMP-9 through the activation of MAPK and ERK1/2 with cancer cell by MMP-9 through the activation of MAPK and ERK1/2 with metabolites FAK and COX-2 [21]. FAK functions as a regulator of MAPK signaling and MMP-9 expression [21]. TNF-α leads to overexpression of COX-2, with hypersecretion of prostaglandins, such as prostaglandin E2, which trigger MMP-9 production by binding to G-protein-coupled receptors, thereby enabling CCA cells to release MMP-9 [21]. EGFR activation via ERK1/2 enhanced focal adhesion turnover via FAK, leading to higher migration speed and fostering of CCA cell motility in addition to inhibiting FOXO4 via activation of PI3K/AKT signaling [50], suggesting the P13K/AKT/mTOR pathway, also reported for HB [11]. Finally, a range of miRNAs, which regulate CCA have been reported in CCA etiology; the upregulated ones include miR34a (target gene: *SMAD4*), miR122 (*RhoA*), miR140-5p (*SEPT2*), miR144 (*PAFAH1B1*/*LIS1*), miR200c (*NCAM1*, *ZEB1/2*), miR204 (*Slug*), miR212 (*FOXA1*), miR214 (*Twist*) and miR605 (*PSMD10*/*gankyrin*) [21]. The downregulated ones include miR21 (*PTEN*, *RECK*, *TIMP3*), miR181c (*NDRG2*) and miR221 (*PTEN*) [21], all offering means for individual biomarkers for diagnosis, therapy and prognosis.

**Table 2:** Metabolites indicated via metabolomics for liver cancers.

Liver Cancer	Metabolites
Hepatoblastoma (HB) ^1^	Lactate, Ammonium, Gammy-glutamyl transferase, Alkaline phosphatase, Methylmalonyl-CoA Mutase MMUT, Methylamlonic acid, Reticulin, Glutamin synthetase, Propionyl CoA, Succinyl CoA, 2-Methylcitrate, Β-catenin expression, Glutamine synthetase expression, Glypican 3 expression, Bile salt export pump expression, Fumarate, Succinate, D-2-Hydroxyglurate (2-HG)
Cholangiocarcinoma (CCA) ^2^	Bile acids (7β), Phophatidylcholine (PC), 2-Hydroxyglurate 2-HG, CXCl12/SDF-1, HMGB1, IL-6, TGF-β, TNF-α, EGF-like family, FGF-19, HGF, Adrenomedullin, Prostaglandin E2, 17β-Estradiol, miRNA, cfDNA, CYFRA 21-1, MMP, osteopontin, periostin, IL-6
Hepatocellular carcinoma (HCC) ^3^	Succinic acid, Fumaric acid, Malic acid, Glucose, Lactic acid, Hypoxanthine, Xanthosine, Adenonsine Monophosphate AMP, Propionylcarnitine, Linoleic acid, Coenzyme Q10, PC (34:2), PC (38:3), PC (36:1), PC (38:2), Glycerol 3-Phosphate, Glycerylphosphorylethanolamine, Glycerophosphocholine, Bile acids, Fatty acids

^1^ Forny et al. [12]; ^2^ Wang et al. [19], Brivio et al. [21] and Amathieu et al. [5]; ^3^ Ferrarini et al. [51] and Amathieu et al. [5] (This list is by no means comprehensive of entire existing literature).

### 3.3. An Integrative Metabolomics Approach to Hepatocellular Carcinoma (HCC)

A wide range of TCA cycle metabolites have been reported for HCC, and in addition for glycolysis, purine and energy pathways [51,52,53], as in the case of HB (refer Section 3.1). A change in the intermediate metabolites of TCA, specifically, succinic acid, fumaric acid and malic acid, have been indicated along with decrease in glucose and increase in lactic acid [52,53] at low aerobic oxidation, thus, at downregulation of coenzyme Q10 [52]. Downregulation of xanthosine and adenosine monophosphate of the purine metabolism were indicated, corroborating that purine metabolism might contribute to cancer progression [54], along with related altered lipid metabolism with reports of downregulation of acylcarnitines [51]. The role of PC was also reported with upregulation of PC (34:2), PC (38:3), PC (36:1) and PC (38:2) [51,52]. Determination of phosphatidylethanolamine-based plasmalogens (PEp) could discriminate HCC patients from healthy controls with association with tumor grading, especially serum Pep (36:4) and Pep (40:6) [55]. Altered fatty acid metabolism in HCC reports enhanced arachidonic acid synthesis along with change in glycerolipid metabolism, especially glycerol 3-phosphate, glycerylphosphorylethanolamine and glycerylphosphocholine; all these metabolites were significantly lower in HCC tumor compared to non-tumor tissues [5,6,7,8,9,10,11,12,13,14,15,16,17,18,19,20,21,22,23,24,25,26,27,28,29,30,31,32,33,34,35,36,37,38,39,40,41,42,43,44,45,46,47,48,49,50,51,52,53,55]. 

Metabolomically, HCC is currently the most investigated among all the liver cancers. Chaudhary and colleagues [56], with deep learning based epidemiological modeling, reported that the aggressive subtype of HCC is associated more frequently with mutations of TP53, higher expression of KRT19 and EPCA and tumor marker of BIRC5 with activated WNT-CTNNB1 and P13K/AKT/mTOR pathways [56]. The role of CHK2 mRNA was reported to be greater in the blood in patients with HCC and CHK2 is known to control the expression of succinate dehydrogenase and mitochondrial functions [53]. High levels of succinate were reported for HB [12,41]. Likewise, high levels of succinate were also reported for HCC and with DNA damage [51,53]. Cells with DNA damage via CHK2 relied on glycolysis for ATP production, due to dysfunctional mitochondria [53]. Dysfunctional mitochondria were negated by CHK2 knockdown, offering new means of therapy in HCC through determination of these metabolites [53]. The pathogenesis of HCC progression was proposed by defects in mitochondrial oxidative phosphorylation and reactive oxygen species (ROS), leading to accumulation of misfolded and unfolded proteins in the mitochondrial matrix, leading to upregulation of mitokines FGF21 and GDF15 along with defects of mitoribosomes, leading to higher ROS in HCC patients [27]. GDF15, indicated in HCC [27], belongs to the TGF-β family, and was also indicated in CCA and cHCC-CCA [21]. Acylcarnitine [51] has been suggested for use in the metabolomics-based diagnosis of HCC as it correlates with tumor grade and was specific in discriminating between HCC and matched normal tissues, and thus, is a potential new candidate for HCC diagnostics and prognostics [52].

## 4. Salient Characteristics of Liver Cancers

The WNT/β-catenin pathway (also WNT-CTNNB1) [30] was reported consistently for HB, CCA and HCC liver cancers [7,15,19,20,30]. Likewise, the P13K/AKT/mTOR pathway was reported consistently for HB, CCA and HCC [11,46,50,56]. This could indicate either a centrality of both these pathways in liver cancer pathophysiology, or that our current understanding of these two pathways is the greatest [11,15]. HB and CCA reported the Hippo pathway, which occurs over the mechanism of IDH1 and IDH2 as well as YAP/TAZ linked to RTKs and GPCRs [7,15,19,20]. CCA was described by mutations in *KRAS* [18,24] and *TP53* [15,24]. Mutations of *TP53* were reported for HCC as well [56]. Among many intermediaries, IDH1/2, BRAF, HER2, PIK3CA, MET, 2HG [15,19,20] were reported for CCA, while FGFR was reported for CCA [15,19,20]. It seems that the RAS-RAF-MEK-MAPK pathway could have a predominant role in HCC [38,39]. Oncometabolite 2-HG and a gene fusion of FGFR2 appeared crucial for CCA [19,20]. 

TCA cycle was downregulated in HB [42] and HCC [51,52,53]. A range of miRNA was reported useful to assay CCA [15,21]. Selenium mitigated oxidative stress via suppression of the NF-kappaB pathway [13]. Dietary fat supplementation and related liver regeneration are under current investigation for HCC; dietary fats are hypothesized to mitigate HCC [30,31,32,33]. Lipid metabolism via acylcarnitine seemed to be crucial in liver cancers [51]. Guri and colleagues reported that mTORC2 promoted de novo fatty acid/lipid synthesis [57]. De novo lipid synthesis upregulated *Sptlc1* expression, which was high in HCC and other tumors. A role of lipids in tumorigenesis was proposed as lipids can function as building blocks for tumor growth, stabilize inner mitochondrial membrane for enhanced energy production by tumor cells, and could function as second messengers in oncogenic signaling pathways, thus in total leading to steatosis and tumor development [57]. The role of PC [52], PEp- [55], Q10 [52], purine metabolism [54] and immune-based regulation via RAS-RAF-MEK-MAPK pathway [35,36,37,38,39] were reported for HCC, which accounts for 80–90% of the adult cases of liver cancer [2].

Both glucose and lipid metabolism were indicated in HCC [29,30,31]. The WNT/β-catenin pathway [30] via ubiquitin ligases [31], which are central to energy metabolism [51,52,53], emerged as one of the central pathways [7,15,19,20,29,30,31]. Ericksen and colleagues [58] reported the suppression of branched-chain amino acid enzymes leading to its accumulation in tumors, which possibly drove chronic activation of mTORC1 [58], indicating that branched-chain amino acid supply and catabolism could be regulating tumor cell proliferation with mTORC1 activity. All three liver cancers reported the P13K/AKT/mTOR pathway [11,46,50,56]. Ali and colleagues [39] proposed a range of natural compounds for various cancers for treatment, such as balcalein, which reduced MEK1, Bad and ERK1/2 in HCC, and Silibinin with sorafenib [28], which reduced phosphorylation of ERK, STAT3, AKT, MAPKp38 [39]. IDH1 and IDH2 related liver cancers of CCA were related to p53, though no mutations were found on p53, but rather hypermethylation [19], indicating epigenetic changes. IDH1/2 associated with overall longer outcome survival and longer time to tumor recurrence [19]. Investigation of biochemical and biophysical metabolomic correlates, such as, the periportal and perivenous oxygen tension [43], could augment current understanding of the links between liver cancers and TCA metabolism with all its metabolites [42,51,52,53]. Nutrition-based schedules comprising of acylcarnitines, short-chain fatty acids, monounsaturated long chain fatty acids, selenium, PC, PEp, Q10, with limitation or alteration of polyunsaturated long-chain fatty acids, branched chain amino acids and glucose [13,27,29,30,31,32,33,43,47,48,51,52,53,55,58] require investigation for prevention, management and treatment of liver cancers. This emerges pertinently, as HB, CCA and HCC are all reported in association with epigenetic regulation in etiology and pathophysiology [10,11,15,19,21]. This would allow disease etiology explanation from the diseases of civilization point of view [2,15,16,17,26,27].

### 4.1. A Proposed Integrative Link between WNT/β-Catenin Pathway and Metabolomics 

**Figure 1:**
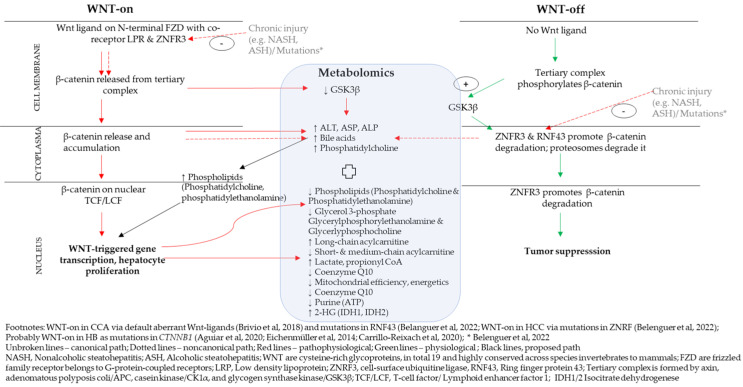
Integrated WNT/β-catenin pathway and metabolomic indicators. References cited in the figure are [3,4,10,21,30].

The WNT/β-catenin pathway probably is the most well-understood and probably well-investigated of all the pathways in liver cancer [7,15,19,20,30]. Figure 1 proposes a link between the WNT/β-catenin pathway with metabolomic indicators. The WNT/β-catenin pathway is explained canonically via Wnt-signal (refer to unbroken lines in Figure 1) [30] and noncanonically via cell-autonomous processes (refer dotted lines in Figure 1), such as systemic modulation via RNF43 and ZNRF3 [30]. Canonically, Wnt ligands bind to FZDs and LRP to suppress activity of GSK-3β. ZNRF3 promotes degradation of WNT receptor function, which is a tumor suppressor signal. With the WNT-on signal, β-catenin associates with TCF/LEF in the cell nucleus and this ensues cell proliferation [30,31,32]. Noncanonically, cell-autonomous processes, as in mutations or chronic injuries, probably including long-term alcohol overconsumption leading to alcoholic steatohepatitis (ASH) or NASH, alters RNF43/ZNFR3 negative feedback control in the WNT/β-catenin pathway (refer Section 2.3) [30,31,32]. Molecular analysis of HCC tumors showed null RNF43/ZNFR3 and high AXIN2 expression [30]. This observation corresponded to an increase in the metabolites of transaminases, ALT, ASP and ALP, all proxies of liver damage [30]. 

Sharif et al. [48] reported higher hepatic bile acid and PC levels in HCC. When RNF43/ZNFR3 function was null, intracellular lipid droplets accumulated in liver and cell differentiation capacity reduced [30]. An increase in triglycerides is required for synthesis of phospholipids, among them PC [52] and PEp-plasmalogens [55]. Furthermore, tumorigenesis requires de novo lipogenesis to supply energy to tumor cells and their proliferation [55]. Higher glycerol-3-phosphate acylation was reported in tumorigenesis [55,57] while systemic glycerol-3-phosphate was low [51]. With no correction of a chronic liver injury status, the lipid accumulation and hepatocyte proliferation and differentiation increased [30]. Faulty liver regeneration with accumulating fibrosis and tissue damage, thus, could probably lead to long-term progression of HCC [30,31,32]. 

A differentiated view on lipid metabolism might be required, though. PC (34:2), PC (38:3), PC (36:1) and PC (38:2) were all upregulated [51,52] and PC (34:2) metabolite was lower systemically [51]. Levels of glycerol 3-phosphate, glycerylphosphoryl-ethanolamine and glycerylphosphocholine were lower in tumor tissues compared to nontumor tissues in HCC, all indicating tumorigenesis [51,52,53,55]. Mutations in RNF43 and ZNRF3 altered lipid metabolism specifically in unsaturated fatty acids, acyl-CoA biosynthesis and non-alcoholic steatohepatitis (NASH) [30]. 

Lu and colleagues [52]. reported higher levels of saturated and monounsaturated fatty acids and low levels of polyunsaturated fatty acids in HCC-tumors [52]. Hepatic cancers show the Warburg effect [52,58,59], that is, a higher rate of aerobic glycolysis with higher lactate in cytosol, suppression of tricarboxylic cycle (TCA), conversion of serine, glycine and threonine into pyruvate, conversion of glutamate to αKG and accumulation of long-chain acylcarnitines and lower short- and medium-chain acylcarnatines [52]. Long-chain acylcarnitines (≥C14) in tumor tissues were used for energy production and to remove short- and medium-chain fatty acids in hepatic mitochondria [51,52]. Thus, short and medium chain fatty acids were low in tumor mitochondria, thus being high in tumors [52]. Normal cells use short- and medium-chain fatty acids in mitochondria for energy production with use of Q10 [51] and propionyl-CoA [41] from fats [52]. Metabolomically, thus, the short- and medium-chain acylcarnitines decreased [52]. The accumulation of propionyl-CoA, which inhibits pyruvate dehydrogenase complex [41], could lower mitochondrial efficiency additionally [41]. 

De novo purine biosynthesis was most efficient when purinosomes were located near to mitochondria [54]. Pedley et al. [54] proposed that purisomes, as metabolons, are formed in response to depleted cellular purine levels (ATP, GTP, NAD, coenzyme A, cell energetics, mitochondrial efficiency) and higher metabolic demands in normal cells. In tumors, mitochondrial reprogramming occurred reducing mitochondrial efficiency [41,54], suggesting tumorigenesis [54]. 

IDH1 and IDH2 mutations were indexed by the increase in oncometabolite 2-HG in CCA [21]. The WNT/β-catenin pathway and P13K/AKT/mTOR pathway indicated 2-HG accumulation in oncogenesis via transcription factor of Sal-like protein 4 (SALL4) [21]. SALL4, which is expressed in fetal hepatoblasts that are under normal conditions, is not expressed by mature heaptocytes (HCC) and cholangiocytes (CCA) [21]. High expression of SALL4 is related to stimulation and expression of Wnt ligands, such as Wnt3a, and promoted liver cancer [21], thereby indicating a default aberrant WNT/β-catenin pathway [21].

### 4.2. A Proposed Integrative Link between P13K/AKT/mTOR Pathway and Metabolomics

**Figure 2:**
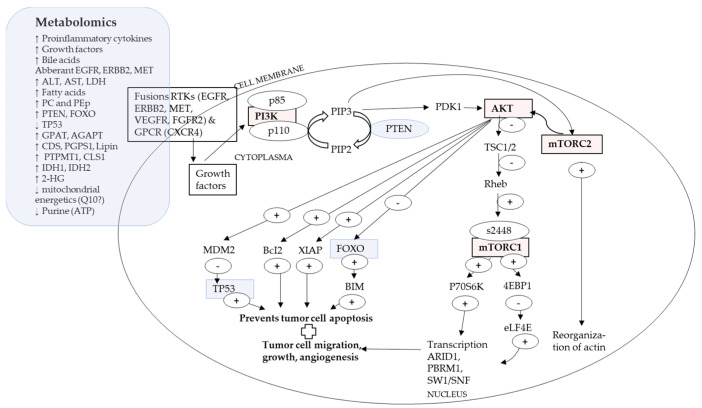
PI3K/AKT/mTOR pathway and metabolomic indicators.

mTOR or mammalian target of rapamycin and its immunosuppression with rapamycin occurs in two forms: mTORC1, which is extremely sensitive to rapamycin, and mTORC2, which is less sensitive to rapamycin (refer to Abbreviations). Inhibition of mTOR with rapamycin caused a decrease in purinosome, a metabolon, and mitochondria colocalization with increased expression of ATF4 [29,54], indicating lower cellular energetics [29]. Concomitantly, the absence of mTOR, due to rapamycin, could suggest lower expression of genes associated with pentose phosphate pathway, which leads to formation of PRPP [11,54], the substrate for the first reaction of de novo purine biosynthesis [54], suggesting lower purine. Figure 2 proposes a link between P13K/AKT/mTOR pathway with metabolomic indicators. 

Receptors RTK or GPCR bind to growth factors, which activates AKT [7,11,46,50,56]. The p110 catalytic subunit of PI3K converts PIP2 into PIP3. PIP3, which activates PDK1, which in turn activates AKT [7,11,46,50,56]. AKT activation leads to phophorylation of mTORC1, which alters modulation of gene transcription, augmenting tumor cell growth, migration, and angiogenesis [7,54]. Likewise, AKT activation leads to phophorylation of mTORC2, which reorganizes actin and leads to higher tumor cell motility [30]. AKT also exerts effects over MDM2, BcI2, XIAP and FOXO, all which lead to suppression of tumor cell apoptosis [11,46,50,56]. Inhibition of FOXO4 was reported in CCA [50]. TP53, a tumor suppressor, is negatively regulated by MDM2 on AKT activation and mutations in TP53 were reported for cHCC-CCA [15,24]. 

Metabolomically, the tumor microenvironment shows higher levels of pro-inflammatory cytokines, growth factors and bile acids [15]. Genomically, there might be vast differences in gene regions while upstream common events may be observed over sub-types [15] and types of hepatic cancers [11], such as a commonality of activation of RTK and GPCR [11,15]. In the embryogenesis phase, hepatocyte differentiation and proliferation depended on function of C/EBPα’s, (Notch signal pathway protein), which binds to SW1/SNF gene, which is one of the key downstream event for P13K/AKT/mTOR pathway [11,46,50,56]. Aggressive liver cancers show upregulation of signaling pathways of mTORC, NOTCH and MYC [15]. The P13K/AKT/mTOR pathway might be a meeting point for the WNT/β-catenin pathway and the Hippo pathway via YAP/TAZ, which are linked to IDH1/2 (refer Table 1) [7].

Liver regulates gluconeogenesis and lipogenesis, e.g., via FOXO1, which in a mutated state, such as in liver cancer, could maintain an undifferentiated cell promotion [7,11]. Upregulation of PTEN, over miR221, was reported in CCA [21]. PTEN under normal conditions functions as a tumor suppressor and suppresses the activation of AKT [7,11,46,50,56]. Serum levels of ALT, AST and LDH were higher in liver cancers [57]. Guri et al. [57] reported that in tumor cells, mTOR promoted de novo lipid and fatty acid synthesis. Fatty acids were further used to de novo synthesize sphingolipids via condensation of serine and palmitate, which, with glycerophospholipids, was required for tumor development [57]. This corresponded to metabolomic high hepatic expression of PC and PEp indicated by enzymes GPAT, AGPAT, Lipin, CDS, PGPS1, CLS1 and PTPMT1 [57]. 

mTORC2 promoted the accumulation of fatty acids, sphingolipids and cardiolipin [57], probably indicating changes in metabolites of lipids, PC, PEp and cell energetics [7,11,29,54]. IDH1 and IDH2 encode metabolic enzymes converting isocitrate and αKG, and thus, mutations in IDH1 and IDH1 lead to accumulation of 2-HG, which is an oncometabolite [15]. Due to hypermethylation of DNA, 2-HG interferes with mitochondrial electron transport chains with low expression of ARID1A, because of epigenetic silencing [15]. IDH1 and IDH2 also lead to multiple chromatin remodeling genes (BAP1, ARID1A and PBRM1) [15], all indicated as probable metabolons and metabolomes that require investigating. 

### 4.3. What Metabolomics Might Offer for the Investigation of Secondary Liver Cancers

Primary liver cancer shows tremendous variability (refer preceding sections). Secondary liver cancer, i.e., a liver cancer that has spread to the liver from another primary index cancer that has started in another part of the body, probably offers even greater variability [59]. The Surveillance, Epidemiology, and End Results (SEER) reported that among 2.4 million cancer patients, 5.14% reported liver metastases [59]. Index cancers were primarily breast cancers for younger women at the ages of 20–50 years followed by a heterogenous population with liver metastases of esophageal, stomach, small intestine, melanoma, bladder, lung, pancreatic, and colorectal cancer, where survival with liver metastases was generally low [59]. Targeting the microbiome at the stomach-gut location indicated that liver cancer is associated with metabolites of hippuric acid, phenylacetylglutamine, phenylacetic acids, benzoic acid, and catechol in gut region [60]. GPCRs (GPCR41, GPCR43 and GPCR109A) can recognize short-chain fatty acids produced by the gut-microbiota [60]. Short-chain fatty acids can regulate gene expression, primarily by inhibiting histone deacetylaseses [60]. Investigation of the variations in production of short chain fatty acids could be of indicative value [60]. In a colon cancer model, the loss of NK cells increased liver metastases, whereas enhanced NK activity reduced liver metastasis [61]. NK underregulation has been also reported in primary liver cancer [34]. Thus, the correspondence of secondary and primary liver cancers with immune suppression emerges as another field warranting research [34,35,36,37,38,39,61]. Liver metastases of breast cancer are the most predominant group [59]. Reports on the measurement of nitric oxide, reactive oxygen species and toxic radicals [59,62,63], released by liver sinusoidal endothelial cells [59], has been proposed [59,62,63]. Measurement of TNF-α has been also suggested [61]. TNF-α has been also indicated in primary CCA [21]. Thus, there appears that certain metabolites might be common to primary and secondary liver cancers, yet differences might emerge in this currently nascent field of research, warranting studies in their own right. 

This current review of HB, CCA and HCC from the viewpoint of epidemiology and cellular studies with interpolation from the growing field of metabolomics encompasses a broad field of literature. Given the vastness of the literature, this review is by no means comprehensive of the entire literature on liver cancers, encompassing molecular physiology up to new-generation epidemiology with deep learning [56]. In this respect, this review remains grossly limited in the vastness and depth of knowledge presentation and analysis. Yet it is an initial attempt to bridge the information gap with metabolomics, to better delineate pathways in a relatively comprehensive manner, over the currently existing literature. There is also a greater requirement to review what emerges as metabolome and metabolon, this review encompassed both. Likewise, it also encompassed known metabolites within metabolomics, yet there might be tremendous differentiation among and within them, which remains for future investigation. There might emerge differences among primary liver cancers of HB, CCA and HCC and the sub-groups of secondary liver cancers as well. This remains a limitation of this current review and deserves future inquiry.

## 5. Conclusions

This review reports that the WNT/β-catenin and P13K/AKT/mTOR pathways were reported in literature for HB, CCA and HCC liver cancers and FGFR changes over all liver cancers. There is a growing body of evidence of the use of metabolomics for diagnosis and therapy of liver cancers, especially with metabolites of TCA cycle, miRNA, IDH1 and IDH2. The role of metabolites such as purine, Q10, lipids, PC and PEp-based plasmalogens, acylcarnitines, 2-HG and propionyl-CoA emerged as crucial. In this review, an initial attempt has been undertaken to link epidemiology and cellular studies via metabolomics over the two pathways of WNT/β-catenin and P13K/AKT/mTOR.

## Figures and Tables

**Table 1:** Primary factors with metabolites in cholangiocarcinoma (CCA) ^1^ pathogenesis.

Genes (Pathways)	Intermediaries/Metabolites	Cell Signal Metabolites
*ARID1*^2^, *PBRM1*, SW1/SNF	STAT3, JAK (JAK/STAT signaling)	IL-6
*ARID1*^2^, PBRM1, SW1/SNF	RAS-MAPK Pathway, KRAS^2^, BRAF, MEK1/MEK2, ERK1/ERK2	EGFR (EGF), ERBB2, MET (HGF), VEGFR (VEGF), FGFR2 fusions, CXCR4 (SDF1)
*ARID1*^2^, *PBRM1*, SW1/SNF, (BAP1)	P13K-AKT, PI3K, AKT, mTOR	EGFR (EGF), ERBB2, MET (HGF), VEGFR (VEGF), FGFR2 fusions, CXCR4 (SDF1)
p300, MAML1, CSL, NCID (Notch pathway)	NCID	NOTCH1-3, JAG1-2, *DLL1*
Β-Catenin, TCF/LEF (WNT/β-catenin pathway)	Β-Catenin, GSK 3β, AXIN, APC, CK1α, DVL, (Ubiquitone)	WNT (LRP5/LRP6)
YAP/TAZ, TEAD (Hippo pathway)	YAP/TAZ, LATS1/2, MOB1, SAV1, MST1/2, Cytoplasm: Citrate, Isocitrate, α-KG, 2-HC via IDH1^2^ (Hippo pathway)	RTKs, GPCRs, (Extracellular matrix stiffness)
YAP/TAZ, TEAD (Hippo pathway)	Mitochondria: Citrate, isocitrate, α-KG, 2-HC via IDH2	-

^1^ As per Banales et al., 2020; ^2^ ARID1A, KRAS, TERT promoter, TP53 and IDH1/2 mutations indicated in cHCC-CCA as per Stavraka et al., 2019 (This list is by no means comprehensive of the entire existing literature).

## Abbreviations

2-HG	D-2-Hydroxyglurate
4EBP1	Eukaryotic translation initiation factor 4E-binding protein
αKG	Alpha Ketoglurate
AGPAT	Acylclycerin-3-phosphate-O-acyltransferase
AKT	Gene AKT1, AKT2, AKT3 for protein kinase B (PKBα/β/γ)
ALT	Alanine transaminase
ALP	Alkaline phosphatase
AST	Aspartate transaminase
AP1	Activator protein 1, a transcription factor
*ARID1A*	AT-rich interaction domain 1A
ATF4	Activating transcription factor 4
BAP1	BRCA1 associated protein 1
Bcl2	B-cell lymphoma 2, a protein, the gene encodes an integral outer Mitochondrial membrane protein that blocks the apoptotic death
*BRCA1/2*	Breast cancer gene 1 and 2
CDS	Diacylglycerol synthases
C/EBPα	CCAAT/Enhancer-binding protein, a Notch signal protein
CHK2	Checkpoint kinase 2, an effector of DNA damage response
CL	Cardiolipin
CLS1	Collagenase, Type 1
COX-2	Cyclooxygenase-2, downstream of TNK-α
CSL	Cotranscriptional factors (such as MAML1, RBPJ, p300)
*CTNNB1*	Catenin beta 1 gene
*CX3CL1/12*	C-X3-C motif chemokine fractalkine ligand 1 gene/ligand 12 gene
*DLL1*	Delta-like canonical notch ligand 1, a protein coding gene
DVL	Disheveled; positive regulator of WNT/β-catenin pathway; DVL is recruited to the plasma membrane and binds to FZD
KEAP1	Kelch-like ECH associated protein 1
*ECT2*	Epithelial cell transforming 2
EGFR	Epidermal Growth Factor-Like Receptor (Family), also known as ErbB1 or HER1
eLF4E	Eukaryotic translation initiation factor 4E
ERBB2	V-erb-b2 erythroblastic leukemia viral oncogene homolog 2
ERK	Extracellular-signal-Regulated Kinase, also known as p44/42
EPCA	Early prostate cancer antigen
FGFR	Fibroblast growth factor
*FGFR2*	Fibroblast growth factor rector 2 gene
FGF21	Fibroblast growth factor 21
FOXO4	Forkhead box protein O4, a protein coded by FOXO4 gene
FUT1	Fucosyltransferase 1
FAK	Focal adhesion kinase, downstream of TNK-α
FZD	Frizzled or class F of G protein-coupled receptors
GDF15	Growth differentiation factor 15 of the family TGF-β
GPAT	Glycerolphosphate-O-Acyltransferase
GPCR	G-protein coupled receptors
GSK 3β	Gylcogen synthase kinase 3 beta
HER2	Human epidermal growth factor receptor, scientific name ERBB2, belongs to HMGB1—High-Mobility Group Box 1 or High-Mobility Group Protein 1, an agent to indicate if cell necrosis has set in
HGF/c-MET	Hepatocyte growth factor-mesenchymal-epithelial transition factor pathway
IDH1/2	Enzyme isocitrate dehydrogenase and the coding genes (*IDH1*gene Arg132, *IDH2* gene Arg172 point mutation)
IL-6	Interleukin 6
JAK	Janus kinase
JAK/STAT3	Janus kinase (JK)—signal transducer and activator of transcription (STAT) pathway
KRT-19	Creatine 19 protein coding gene
LATS1/2	Large tumor suppressor kinase ½ (Hippo pathway, also indicated in WNT/CTNNB1 pathway)
LDH	Lactate dehydrogenase, an enzyme
LGR4-5-6	Leucine-rich repeat-containing G-protein-coupled Receptor (4-5-6)
Lipin	A phosphatase, Overproduction of it promotes obesity
LRP	Low-density lipoprotein Receptor-related Protein (5/6)
LncRNA	Long noncoding RNA
Lnc-EGFR	Long noncoding RNA, specifically the long noncoding epidermal growth factor receptor
MAML1	Mastermind-like transcriptional coactivator 1 (of the Notch pathway, with *NOTCH1*, *MAML1*, *RBPJK* (suppressed upon *GPR50* knockdown))
MAPK	p38 Mitogen-activated protein kinase
MDM2	Mouse double minute 2 protein coding gene, a proto-oncogene; The gene encodes a nuclear-localized E3 ubiquitin ligase, the encoded protein can promote tumor formation, essential for p53 regulation
MET	Protein oncogene, also called c-Met or tyrosine protein kinase Met or hepatocyte growth factor receptor (HGFR)
MMP-7/9	Matrix metalloproteinase with zinc-dependent endopeptidases, and a protein coding gene
MOB1	Monopolar spindle-one-binder protein (1)
MST1/2	Mammmalian STE20-like protein kinase 1/2, regulate lymphocyte development, trafficking, survival and antigen recognition by naïve T cells, MST1/2 and YAP expression increased in platelet-driven growth factor receptor-α 8PDGFRα)
mTOR	Mammalian target of rapamycin, immunosuppression with rapamycin
mTORC1	mTOR complex 1, made up of mTOR, Raptor, mLST8, PRAS40, extremely sensitive to rapamycin
mTORC2	mTOR complex 2, made up of mTOR, Rictor, Sin1, mLST8, a serine/threonine kinase mTOR less sensitive to rapamycin
MUC1/4	Mucine 1/4 (Protein)
NCID	Intracellular domain of Notch
NFE2L2	Nuclear factor, erythroid 2 like bZIP transcription factor 2
NFE2L2/KEAP1	Nuclear factor, erythroid 2 like bZIP transcription factor 2- Kelch-like ECH associated protein 1 pathway
NF-kappaB	Nuclear factor kappa-light-chain-enhancer of activated B-cells protein complex
NK	Natural killer cells, of the immune system
p300	Histone acyltransferases, such as, p300 (others: CBP, SAGA, etc.)
P72S6K	Ribosomal protein S6 kinase beta-1
*PBRM1*	Polybromo 1 gene
PC	Phosphatidylcholine (a kind of phospholipid)
PCK 1	Phophoenolpyruvate carboxykinase 1
PDC	Pyruvate dehydrogenase complex
PDK1	Phosphoinositide-dependent kinase-1
PEp	Phosphatidylethanolamine/plasmalogen (a kind of phospholipid)
PGPS1	Phosphatidylglycerophosphate synthase 1
PERK	Protein kinase R (PKR)-like endoplasmic reticulum kinase
P13K/AKT/mTOR	Phosphoinsositide-3-kinase (PI3K)/protein kinase B (AKT) (of AGC kinase family)-/mammalian target of rapamycin (mTOR) pathway, PI3K is an heterodimeric enzyme protein involved in lipid synthesis composed of large family of lipids and serine/threonine kinases and has p110 catalytic and p85 regulatory subunits
PIK3CA	Phophatidylinsositol-4,5-bisphosphate 3-kinase catalytic subunit alpha (a protein coding gene)
*PLK1*	Polo like kinase 1
*PRKCA/PRKCB*	Protein kinase C alpha/beta
PIP2	Phosphatidylinositol 4,5-bisphosphate
PIP3	Phosphatidylinositol 3,4,5-triphosphate
PRPP	Phosphoribosylpyrophosphate
PTEN	Phosphatase and tensin homolog, a tumor suppressor protein, which suppresses activation of AKT
PTPMT1	Mitochondrial phosphatase 1
RAF	Serine/Threonine protein kinase, e.g., ARAF, BRAF and CRAF (refer to SMAD)
RAS	Renin angiotensin system
RAS/ERK/AP1	Renin angiotensin system-Extracellular-signal-regulated kinase-Activator protein 1 pathway; also RAS/RAF/MEK/MAPK activated RAS-a small G-protein-activated BRAF-activated MEK-activated ERK-MAPK
Rheb	Ras homolog enriched in brain, a GTP-binding protein
RNF43	Ring Finger Protein 43 (a protein coding gene)
RSPO2	R-Spondin 2 (a protein coding gene)
RTKs	Receptor Tryrosinkinases (also RYKs)
SAV1	Salvador fmailiy WW domain containing protein 1
SDF-1	Stromal cell-derived factor 1
SMAD	Protein and genes, a tumor suppressor gene *SMAD* and function as Main signal transducer for receptors of the transforming growth factor, activated by protein serin/threonine kinase (refer to RAF)
SP/NK-1R	Substance P and the neurokinin-1 receptor
SPTLC1	Serine Palmityoltransferase, long chain base subunit 1, a protein Encoded by *SPLTC1* gene
STAT3	Signal transducer and activator of transcription 3
SW1/SNF	SWitch/Sucrose Non-Fermentable, subfamily of ATP-dependent chromatin remodeling complexes
TCF/LEF	T-cell factor-lymphoid enhancer-binding factor transcription Factors
TEAD	WWTR1/TAZ-transcriptional enhanced associate domain
TERT	Telomerase-reverse-transcriptase
TGF-β	Transforming growth factor-β, a pleiotropic cytokine three forms TGF-β1, TGF-β2, TGF-β3) (see GDF15)
TNF-α	Tumor necrosis factor-α, a pleiotropic cytokine belonging to the TNF superfamily, predominantly expressed by macrophages, T and B lymphocytes, and natural killer (NK) cells
TSC1/2	Tuberous sclerosis complex 1 and 2
TP53	Protein 53
VEGF	Vascular endothelial growth factor
VEGFR	VEGF receptor
WNT	Wingless-related integration site (identified first in *Drosophila*)
WNT/β-catenin	*WNT* gene- β-catenin pathway, the canonical WNT pathway Involves nuclear translocation of β-catenin and activation of target genes via T-cell factor/lymphoid enhancer-binding factor (TCF/LEF) transcription factors, controlling cell proliferation The noncanonical WNT pathway is independent of β-catenin and TCF/LEF (e.g., WNT/Ca^2+^ pathway) and regulates cell polarity and migration. Both WNT pathways are mutually regulated.
XIAP	X-linked inhibitor of apoptosis protein
YAP/TAZ	Transcriptional regulators YAP (Yes-associated protein 1) and TAZ
ZNRF3	Cell-surface transmembrane E3 ubiquitin ligase zinc and ring finger (a protein coding gene, which with RNF43 and RSPO2 are considered as a master switch that determines specification by similarity)

## Appendix A

**Table A1:** Common genes reported in hepatic steatosis and hepatic cancers.

Steatosis ^1^	Cancers ^2^
	*AFP*
*ALDH3A2*	
*ALDH7A1*	
*ALDOC*	
*ANGPT4*	
	*ARID1A*
	*ATF4*
	*ATM*
	*AURKB*
	*AXIN1*
	*AXIN2*
	*BAP1*
	*BEX1*
	*BICCI1*
	*BIRC5*
*BRAF*	*BRAF*
	*BRCA1*
	*BRCA2*
	*BUB1*
	*CCND1*
	*CCND2*
	*CDC2*
*CREB3L4*	
*CRTC2*	
	*CTNNB1*
	*CYP (CYP1A1, FYP2E1)*
	*DLG7*
	*DLK1*
	*ECT2*
	*EGFR*
*EFNA1*	
	*ERBB1-3*
*FGF12*	
*FGFR4*	*FGFR2,* FGFR*1-3*
	*GLUL*
*GPI*	
*GNA13*	
	*HDAC2*
	*IGF2*
*IKBKG*	
*INSR*	
*ITGAV*	
	*KDM*
	*KMT2C*
	*KRAS*
*MAP2K2*	
*MAP3K7*	
	*METLL1*
	*MEG3*
	*MGEA5*
	*MML*
	*MYC*
	*NDN*
*NFKB1*	
	*NPM1*
*OGT*	
	*PBRM1*
*PDGFB*	
	*PEG3*
	*PEG10*
*PFKL*	
*PHKA1*	
*PHKB*	
	*PIK3CA*
	*PPHLN1*
	*PLK1*
*PPP1CA*	
*PRKACA* *(CAMP activated)*	
	*PRKCA-PRKCB* *(activated Ca & Diacylglycerol)*
	*PTEN*
	*RHBG*
	*RNF43*
*RRAS2*	
*RPS6KA2*	
	*SMAD4*
	*SMARCA*
	*STK11*
	*TACC3*
	*TACSTD1*
*TAPBP*	
	*TP53*
*VEGFB*	
	*ZNRF3*

^1^ As per Hoyles et al., 2018; ^2^ Text in Sections of “Current Understanding” light grey boxes for HB; white boxes for CCA and dark grey boxes for HCC (This list is by no means comprehensive of the entire existing literature).
